# Left Main PCI, Still a Main Issue

**DOI:** 10.5812/cardiovascmed.14740

**Published:** 2013-10-28

**Authors:** Farshad Shakerian

**Affiliations:** 1 Interventional Cardiololy Department, Shahid Rajaie Cardiovascular Medical and Research Center, Iran University of Medical Sciences, Tehran, IR Iran

**Keywords:** Left main, Stenting, Drug- Eluting Stent

In this issue of journal, we have an observational study on left main PCI, a hot debate in interventional cardiology field (1). They reported that MACCE rate of 22% in one year follow-up seems to be pretty high. Several flaws make the interpretation of the results difficult. First and foremost, how could you mix protected and unprotected LM stenting together? Most of the unprotected ones didn’t have a better option, but protected LM stenting should be compared with other options: the good old CABGs. As far as I’m concerned, most of unprotected LM PCIs were done because of LM dissection or emergent conditions which make the results even more complicated. Using so many bare metal stents in this significant place again shows the emergency situation of the condition which was not stated. One year follow-up is not enough but still the MACCE rate is high. I mean if we had longer follow-up, higher figures where expected (2-4). Considering descriptive type of the study and mixing so many different types of patient subgroups which are actually completely different (protected and unprotected, emergent & elective, BMS & DES) makes the results uniterpretable. Using BMS in LM is not ethical in elective cases and I think they have used them in emergency situations. This further implies the inability to compare results (5, 6). Protected LM (includes most cases which is completely another issue and putting it together with unprotected cases is mixing two mutually exclusive issues). Although LM atherosclerosis seems to be responsive to risk factor modification and more or less under influence of the same factors as other vessels are, it has some different characteristics (7). The main difference is the presence of a large side branch (if we could call Left circumflex a side branch!) which makes the most interventions on LM, a bifurcation lesion. As most of bifurcations happens, ostium of circumflex shows the highest rate of restenosis unless you use two stents which makes the procedure cumbersome (8, 9). In the end, I would like to stick to the latest RCTs and do recommend PCI for low risk anatomy with good SYNTAX score and surgery for the others.
